# Oscillometry for the diagnosis of asthma in children: a systematic review

**DOI:** 10.1183/16000617.0293-2025

**Published:** 2026-04-29

**Authors:** Senali Y. Seneviratne, Saxon Law-Gleen, Jonathan Broomfield, Pip Divall, Pooja Devani, Francine M. Ducharme, Erol A. Gaillard

**Affiliations:** 1 Department of Respiratory Sciences, Leicester NIHR Biomedical Research Centre (Respiratory theme), University of Leicester, Leicester, UK; 2 Nottingham University Hospitals NHS Trust, Nottingham, UK; 3 Department of Population Health Sciences, University of Leicester, Leicester, UK; 4 University Hospitals of Leicester NHS Trust, Leicester, UK; 5 Departments of Paediatric and of Social and Preventive Medicine, University of Montréal, Montréal, QC, Canada; 6 F.M. Ducharme and E.A. Gaillard share senior authorship

## Abstract

**Background:**

Diagnosing asthma in children and young people (CYP) remains challenging. Oscillometry is a promising tool and is feasible from 2 years of age. European Respiratory Society (ERS) technical standards and bronchodilator response (BDR) oscillometry thresholds have been published, but diagnostic accuracy is not established.

**Methods:**

We systematically reviewed studies comparing oscillometry and spirometry in CYP under investigation for asthma. Reference standards were positive BDR or positive methacholine challenge test (MCT). Primary aims were to investigate the sensitivity and specificity of current ERS oscillometry thresholds (>40% decrease in resistance at 5 Hz (*R*_5_), >50% increase in reactance at 5 Hz (*X*_5_) or >80% decrease in the area under the reactance curve); secondary aims were to identify oscillometry threshold values optimising both sensitivity and specificity.

**Results:**

11 studies were included; six (n=992 CYP) utilised BDR and five (n=531 CYP) MCT as reference standard. Meta-analysis was not possible due to heterogeneity of results reported. In two studies using current ERS BDR thresholds, zero sensitivity and high specificity (>85%) were observed. In weighted regression analyses of BDR studies, a 17.0% decrease in resistance at 5–6 Hz had sensitivity and specificity of 71.6% (95% CI 69.7–73.7%); a 20.2% increase in *X*_5_ had sensitivity and specificity of 68.6% (95% CI 66.6–70.8%). Similarly, 27.7% increase in *R*_5_ had sensitivity and specificity of 73.6% (95% CI 71.9–75.3%) for MCT.

**Conclusion:**

Currently recommended ERS thresholds for oscillometry BDR have low sensitivity. Proposed thresholds for defining positive BDR and MCT by oscillometry require prospective validation and adoption of standards for measuring and reporting oscillometry parameters in future diagnostic comparative studies.

## Shareable abstract

**European Respiratory Society recommended oscillometry thresholds to diagnose asthma following bronchodilation have low sensitivity and future threshold studies require adoption of standards for measuring and reporting oscillometry parameters.** https://bit.ly/4r1vx4W

## Plain language summary

Asthma is a common breathing disorder and is difficult to diagnose, particularly in young children. Oscillometry is a simple breathing test that is quick, painless and can be done from age 2 years onwards. The European Respiratory Society (ERS) recently provided oscillometry cut-off values to use for diagnosing asthma but the accuracy of these values is not known. Our review paper evaluates the accuracy of oscillometry for diagnosing asthma in children.

We included studies testing oscillometry in children (2–18 years) investigated for asthma. We compared oscillometry to two well-established spirometric tests, namely “bronchodilator response” (BDR) and “methacholine challenge test” (MCT). Our aim was to examine how accurately oscillometry could diagnose asthma in these children compared to the well-established tests.

We included 11 studies in our review. Six studies compared oscillometry to spirometry with BDR. Two of these studies used the cut-off values recommended by ERS; they showed that these cut-off values were likely to cause a high proportion of “false-negative” results. We analysed the results of the four remaining studies, which all measured the same oscillometry parameter; we identified an alternative cut-off value that maximised sensitivity and specificity equally.

The remaining five studies in our review compared oscillometry to MCT. We analysed the results of three studies and identified an alternative, balanced oscillometry cut-off value for diagnosing asthma.

In summary, the currently recommended BDR cut-off values for oscillometry are likely to result in too many “false-negative” test results (*i.e.* children who have asthma but are not being diagnosed correctly). Based on our analyses, we suggest alternative, more balanced cut-off values that maximise sensitivity and specificity equally; these still need to be validated in future studies. Additionally, we recommend that all future studies evaluating oscillometry measure and report the same parameters to allow aggregation of results.

## Introduction

Asthma is the most common chronic respiratory condition affecting children and young people globally [1]. It is associated with significant morbidity and mortality [2] and places a huge burden on healthcare resources [3]. Diagnosing asthma without objective tests leads to significant rates of misdiagnosis, including both under- and overdiagnosis [4]. In contrast, performing objective lung function tests has been shown to reduce the risk of severe asthma attacks [5] and improve adherence to asthma controller medication [6, 7]. A recent European Respiratory Society (ERS) task force recommended spirometry with bronchodilator reversibility (BDR) testing and fractional exhaled nitric oxide (*F*_ENO_) as first-line tests for all children aged 5 years or older under investigation for asthma. Objective diagnostic tests are also recommended by the Global Initiative for Asthma (GINA) [8] and the UK National Institute for Health and Care Excellence, the Scottish Intercollegiate Guidelines Network and the British Thoracic Society [9].

However, diagnosing asthma using currently recommended first-line tests is challenging because the tests are frequently unavailable due to lack of access or training and, when available, BDR test results are nondiagnostic in the majority of children tested [10–12]. Not all school-age children can manage spirometry with BDR and *F*_ENO_ [10–12] and, particularly, children under 5 years of age struggle to reproducibly perform the forced expiratory manoeuvres required for spirometry, peak flow or *F*_ENO_ measurements. Thus, there are no recommended tests for children under 5 years of age, representing a major unmet need.

Wheezing is highly prevalent in young children with approximately one-third having had at least one wheeze episode by 3 years of age [13–15]. Preschoolers also have the greatest burden of acute severe wheeze exacerbations of all age groups [16] and they are often poorly managed [17]. Approximately 50% of all children with preschool wheeze will go on to receive a formal diagnosis of asthma during school age [18–22]. Poor disease control in the first 2 years following a diagnosis of preschool asthma is associated with increased likelihood of persistent asthma at school age poor long-term symptom control and increased healthcare use throughout childhood and adolescence. Thus, prompt, accurate diagnosis of asthma in preschool children is essential to ensure they are started on appropriate treatments early to improve their symptom control.

Portable oscillometry, a quick and noninvasive lung function test, is effort-independent, performed during quiet tidal breathing and requires minimal patient co-operation. The approach has been shown to be feasible in young children, including preschoolers [23, 24]. Oscillometry superimposes external soundwaves over one or a range of frequencies on spontaneous tidal breathing to measure respiratory impedance [25]. Impedance is then separated into two key parameters, resistance (*R*) and reactance (*X*). Oscillometry has been used to assess BDR as well as airway hyperresponsiveness with bronchial provocation testing.

The ERS recently published technical standards for respiratory oscillometry and thresholds for BDR derived from healthy children [26]. They suggest defining a positive oscillometric BDR when one of the following thresholds are met: >40% decrease in resistance at 5 Hz (*R*_5_) or >50% increase in reactance at 5 Hz (*X*_5_) or >80% decrease in A*X* (area under the reactance curve) [26]. However, the sensitivity and specificity of oscillometry as a diagnostic tool in children suspected of asthma has not been definitively established, nor have the latest ERS thresholds been evaluated specifically in this population. Consequently, oscillometry is not currently recommended as a diagnostic test for asthma by any clinical guideline. We conducted a systematic review to evaluate the sensitivity, specificity and diagnostic accuracy of oscillometry as an asthma diagnostic test in children and young people, including preschoolers.

## Methods

We conducted a systematic review to evaluate the diagnostic accuracy of oscillometry compared to that of spirometry with BDR and MCT in children and young people, including preschoolers, under investigation for asthma.

The Preferred Reporting Items for a Systematic review and Meta-Analysis of Diagnostic Test Accuracy studies (PRISMA-DTA) checklist [27] was used to guide this systematic review. This review was registered *a priori* in the international prospective register of systematic reviews, PROSPERO (registration number CRD42023452091).

### Search strategy

Medline, Embase, EMCare, Cumulative Index to Nursing and Allied Health Literature (CINAHL) and Cochrane Central Register of Controlled Trials (CENTRAL) databases were searched from 1946 to 12 March 2025 using a comprehensive search strategy developed in collaboration with an experienced librarian (P. Divall) (supplementary appendix 1).

### Inclusion criteria

Inclusion criteria for scientific publications for this review were as follows.

#### Study design

Longitudinal, cross-sectional or cohort studies evaluating diagnostic accuracy of oscillometry.

#### Population

Children aged 2–18 years under investigation for asthma, recruited from outpatient clinic settings. We intentionally decided to include children under investigation for asthma as this is the exact population to be targeted for the development of a new diagnostic test.

#### Index test

Respiratory oscillometry carried out at any single frequency or over a range of frequencies, in line with recommended technical standards [26]. Studies evaluating pre-specified or exploratory diagnostic thresholds were considered.

#### Reference standard

Diagnostic reference standards, measured by spirometry including either 1) a positive bronchodilator response (BDR), defined as a ≥12% increase from baseline in forced expiratory volume in 1 s (FEV_1_), or 2) a positive methacholine provocation test, defined as a ≥20% decrease in FEV_1_ after a pre-specified dose of methacholine within the accepted range of 4–16 mg·mL^−1^ or equivalent.

#### Outcome metrics

Primary outcome metrics were sensitivity and specificity of oscillometry parameters for defining positive BDR or MCT. The secondary outcome metric was area under the receiver operating characteristic curve (AUROC).

### Exclusion criteria

Articles were excluded if they were not published in full text (*e.g*. conference abstract only), not published in English or not primary research studies (*e.g.* reviews, editorials, case reports or case series).

### Study selection and data extraction

Search results from all databases were compiled and duplicate results removed using SRAccelerator [28]. Two reviewers (S.Y. Seneviratne and S. Law-Gleen) independently screened the remaining titles and abstracts, manually removed any remaining duplicates, and identified studies for full-text review. They then independently reviewed the full-text versions for eligibility. Any disagreements between reviewers at either stage were resolved through discussion. If they could not reach an agreement, a third reviewer (P. Devani or E.A. Gaillard) acted as arbitrator. Pre-specified data were extracted from full-text articles by one reviewer (S.Y. Seneviratne) and independently verified by a second (S. Law-Gleen).

### Definitions for data extraction

Data extracted from studies included demographic information of participants (including age, gender and height), oscillometry devices, oscillatory frequencies and signals used, spirometry devices used, quality control measures applied for both oscillometry and spirometry measurements, as well as doses of bronchodilator or methacholine used. Extracted oscillometry measurements included resistance at key frequencies (*i.e.* 5 Hz (*R*_5_), 6 Hz (*R*_6_), 8 Hz (*R*_8_), 10 Hz (*R*_10_) and 20 Hz (*R*_20_)), *X*_5_ and A*X*. Extracted spirometry measurements included FEV_1_. Oscillometry and spirometry measurements were extracted pre- and post-bronchodilator or pre- and post-provocation testing, as indicated.

Sensitivity, specificity and AUROC were directly extracted from studies where possible or calculated if not (see statistical analyses). For studies published within the last 15 years, in which data required to calculate key outcome metrics (sensitivity and specificity) was missing or incomplete, corresponding authors were contacted *via* email with one follow-up reminder. Authors of all included studies were contacted to request their raw data to allow us to calculate receiver operating characteristic (ROC) curves, if not already published.

### Risk of bias assessment

All studies included in the review were assessed for their risk of methodological bias and concerns regarding applicability independently by two reviewers (S.Y. Seneviratne and S. Law-Gleen). Analysis using the Quality Assessment of Diagnostic Accuracy Studies (QUADAS-2) [29] tool evaluated the risk of bias within four domains, namely patient selection, index test, reference standard and flow and timing. Each included study was rated as being at high, low or unclear risk of bias within each domain. Concerns regarding applicability were assessed within three domains, namely patient selection, index test and reference standard, and were classified as high, low or unclear for each study. Any disagreements between the two reviewers were resolved through discussion, with a third reviewer (P. Devani or E.A. Gaillard) acting as arbitrator if required.

### Statistical analyses

Statistical analyses were carried out using GraphPad Prism version 10.0.0 (GraphPad Software, Boston, Massachusetts, USA) and Stata version 18.0 (StataCorp, College Station, Texas, USA). Statistical significance was defined as p<0.05.

Sensitivity, specificity and AUROC were directly extracted from studies where possible. When these values could not be directly extracted, 2×2 contingency tables were created based on published data or data provided by authors upon request and analysed using two-sided Fisher's exact test to generate 95% confidence intervals. We initially aimed to conduct a meta-analysis to provide a summary estimate of the sensitivity and specificity of oscillometry parameters for defining a positive BDR or positive MCT. *Post hoc*, we conducted a weighted linear regression analysis that modelled the relationship between the oscillometry threshold proposed by individual studies and the corresponding sensitivity and specificity to determine optimal threshold values for oscillometry parameters. The weights were proportional to each study size, calculated using an inverse of variance. The model fit was assessed using residual diagnostic plots (comparing to fitted values and assessing normality). As sensitivity and specificity are inversely correlated, a bivariate regression model was considered for each group to account for this relationship. As different diagnostic thresholds led to an increase in sensitivity (or specificity) and a decrease in the other parameter, the primary purpose of the model was to identify the crossover point where the two parameters were equally maximised. Thus, we defined the “optimal threshold” as the threshold at which sensitivity and specificity were equal. Multiple approaches for determining an “optimal” threshold exist, depending on the clinical context of whether sensitivity (ruling in a disease) or specificity (ruling out a disease) is more important. In addition to our approach of jointly maximising both sensitivity and specificity, we also conducted analyses based on the product (maximum product of sensitivity×specificity), Youden index (maximum value of sensitivity+specificity−1) and 95% specificity approaches [30].

Additional *post hoc* analyses included a sensitivity analysis excluding studies at high risk of bias from the above weighted regression model and a subgroup analysis evaluating the effect of dose of bronchodilator used for BDR testing.

## Results

**FIGURE 1:**
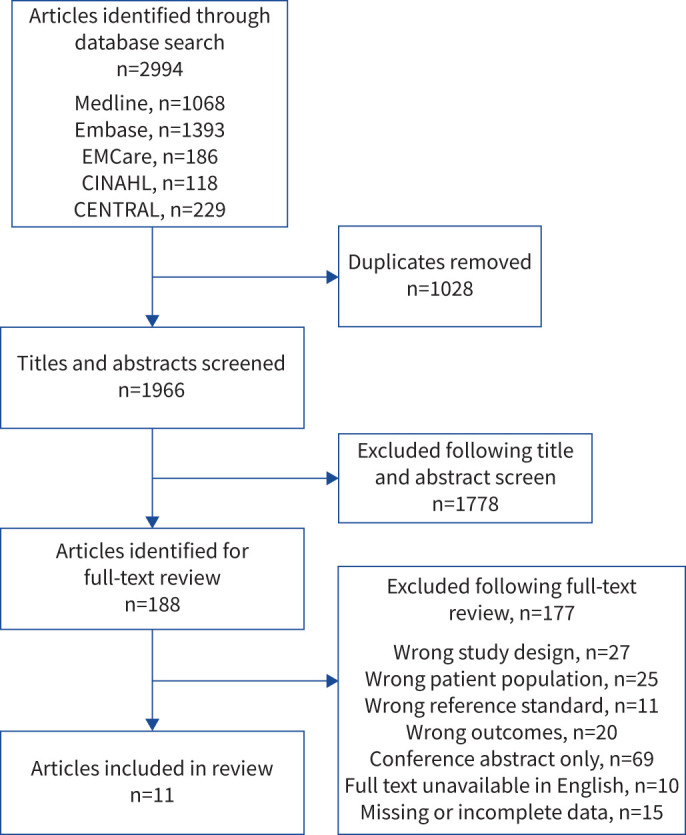
PRISMA (Preferred Reporting Items for a Systematic review and Meta-Analysis) flow diagram for the systematic review.

The search strategy yielded a total of 2994 articles with 1966 articles remaining following the removal of duplicates. Following a title and abstract screen, 188 articles were selected for full-text review; 177 articles did not meet inclusion criteria, resulting in 11 included studies. Six studies utilised spirometry with BDR as the reference standard whilst five studies utilised MCT as reference standard. The PRISMA flow diagram is shown in figure 1.

### Risk of bias

**FIGURE 2:**
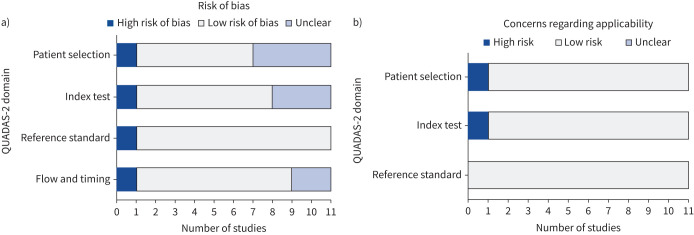
Summary of the a) risk of bias and b) concerns regarding applicability for included studies assessed using the QUADAS-2 (Quality Assessment of Diagnostic Accuracy Studies 2) tool [29].

Using the QUADAS-2 tool [29], eight of 11 studies were rated as being at low or unclear risk of bias within all four domains. 10 of 11 studies were at low risk of concerns regarding applicability in all three domains. The results of our analysis using the QUADAS-2 tool [29] are summarised in figure 2 with additional details in table S1.

#### Patient selection

Six studies included only children under investigation for asthma and were deemed to be at low risk of bias as this is our target population. Four studies included children with asthma and controls who had other respiratory diagnoses. These studies were deemed to be at unclear risk of bias as it was uncertain how nonasthma respiratory conditions would affect participants’ spirometry and oscillometry results. Finally, Jara-Gutierrez *et al.* [31] included participants under investigation for asthma with a small proportion of age-matched healthy controls; it was deemed to be at high risk of bias because comparing oscillometry results of participants under investigation for asthma to those of healthy controls might overestimate the sensitivity and specificity of oscillometry.

#### Index test

Seven studies described in detail the protocol for acquiring oscillometry measurements including patient positioning with appropriate cheek support, replicate measurements being taken and the coefficient of variation (CV) or coherence of measurements being evaluated to ensure quality control of measurements. One study (Bar-Yishay *et al.* [32]) reported that a minimum of one acceptable measurement was used using short acquisition times (8 s); this study was deemed to be at high risk of bias for not meeting recommended ERS technical standards of three replicate measurements with CV ≤15% for resistance at low frequencies [26]. The remaining three studies did not clearly describe the quality control measures utilised when measuring the index test and were thus deemed at unclear risk of bias.

#### Reference standard

10 of 11 studies adequately described detailed methods for assessing the reference standard including using a minimum of three acceptable spirometry measurements. Bar-Yishay *et al.* [32] utilised a minimum of one acceptable spirometry measurement and was deemed to be at high risk of bias.

#### Flow and timing

10 studies specified that oscillometry was carried out prior to spirometry measurements, as recommended. Bouaziz *et al.* [33] reported measuring oscillometry and spirometry in a pseudorandom order and was thus deemed to be at high risk of bias. Nine studies reported that participants’ asthma preventer therapies were withheld prior to involvement in the study. Short-acting bronchodilators were withheld for at least 6 h whilst long-acting bronchodilators were withheld for at least 12 h. Bar-Yishay *et al.* [32] and Lauhkonen *et al.* [34] did not ask participants to withhold these medications and were deemed at unclear risk of bias for this domain.

### Applicability of findings

All participants were recruited from outpatient clinics. Bouaziz *et al.* [33] did not report participant height, which is known to influence oscillometry and spirometry measurements, and was deemed to be at high risk of concerns regarding applicability. Eight of 11 studies did not report participant ethnicities, but there is currently no evidence to suggest that ethnicity affects respiratory oscillometry values and the Global Lung Function Initiative recommend the development and use of race-neutral thresholds [35]. Thus, this did not affect our assessment of the applicability of findings from these studies.

All studies used commercially available spirometry and oscillometry devices. Bouaziz *et al.* [33] used a nonportable oscillometry device where oscillations were applied to a head generator enclosing the participant's head. This system is quite different from currently available portable oscillometry devices; thus, this study was deemed to be at high risk of concerns regarding applicability. There were no concerns regarding applicability of the reference standard from any included studies.

### Studies evaluating BDR

Six studies evaluated the diagnostic accuracy of oscillometry compared to spirometry with BDR as reference standard for the diagnosis of asthma.

#### Study characteristics

Studies included only participants under investigation for asthma except for Bar-Yishay *et al.* [32] and Sheen *et al.* [36], which also included a small proportion of participants with other respiratory diagnoses. Sheen *et al.* [36], Bar-Yishay *et al.* [32] and Lauhkonen *et al.* [34] carried out oscillometry measurements at single frequencies only (5 Hz, 6 Hz and 8 Hz, respectively), whilst the remaining three studies carried out oscillometry over the 5–20 Hz frequency range.

Most studies reported adherence to data acquisition and reproducibility criteria for quality control (table S2). Notably, only Ramirez *et al.* [37] and Meoli *et al.* [38] evaluated the reproducibility of measurements using CV. Only Lauhkonen *et al.* [34] and Meoli *et al.* [38] reported z-scores for oscillometry values, which is recommended by the ERS technical standards [26]. Bar-Yishay *et al.* [32], Ramirez *et al.* [37] and Meoli *et al.* [38] analysed and reported the percentage of predicted normal values for oscillometry parameters whilst Komarow *et al.* [39] and Sheen *et al.* [36] analysed and reported raw values.

The reference standard for all studies was a change in baseline FEV_1_ of 12% or more. Three studies utilised a salbutamol dose of 200 µg or equivalent for assessing bronchodilator response and three utilised 400 µg (table S2).

#### Analyses and results

Demographics of study participants along with reported thresholds for oscillometry parameters and their associated sensitivities and specificities are summarised in table 1. Considering the feasibility of BDR testing, 864 of 992 participants (87.1%) across all six studies were able to complete both spirometry and oscillometry. Bar-Yishay *et al.* [32] and Meoli *et al.* [38] included the youngest study participants, with approximately half of participants able to complete both spirometry and oscillometry (table 1).

**TABLE 1:** Results of studies evaluating spirometry with bronchodilator response (BDR)

First author [ref.], year	N (% male)	n (% male)	Positive BDR by spirometry (n (%))	Age (years)	Height (cm)	Oscillometry parameter and cut-off value	Sensitivity % (95% CI)	Specificity % (95% CI)	AUROC
**Bar-Yishay [32] 2009**	46 (NR)	24 (NR)	6 (25.0)	Median (range) 4.9 (1.8–18.3)	Median (range) 109 (81–163)	>28% decrease in *R*_6_	83.3 (43.7–99.2)^¶^	83.3 (60.8–94.2)^¶^	–
**Komarow [32] 2012**	117 (56.4)	82 (NR)	66 (80.5)	Group A: 7.7*±*3.6 Group B: 7.3*±*4.2	Group A: 126.4*±*19.9 Group B: 126.8*±*25.5	>11.2% decrease in *R*_5_	73	66	0.65
						>8.6% decrease in *R*_10_	77	76	0.72
						>5.9% decrease in *R*_20_	62	65	–
						>18.2% increase in *X*_5_	59	69	0.66
						>29.1% decrease in A*X*	67	69	0.66
**Sheen [36] 2018**	592 (NR)	575 (58.7)	149 (25.9)	Median (IQR) 6.4 (5.1–9.3)	Median (IQR) 120 (109–134)	>34% decrease in *R*_5_ or >0.29 kPa·L^−1^·s^−1^ absolute decrease in *R*_5_ or >50% increase in *X*_5_ or >0.18 kPa·L^−1^·s^−1^ absolute increase in *X*_5_	32.2 (25.2–40.1)^¶^	88.5 (85.1–91.2)^¶^	–
**Lauhkonen [34] 2021**	72 (NR)	54 (51.8)	20 (37.0)	11.6*±*3.3^#^	146*±*17.4^#^	>32% decrease in *R*_8_	45.0 (25.8–65.8)^¶^	94.1 (80.9–99.0)^¶^	–
**Ramirez [37] 2021**	93 (60.2)	93 (60.2)	13 (14.0)	Median (IQR) 11 (8–13)	Group a: 149.0 (127.5–152.0) Group b: 146.5 (134.7–156.0) Group c: 142.0 (127.5–155.0)	>40% decrease in *R*_5_	0.0 (0.0–20.4)^¶^	98.7 (93.1–99.9)^¶^	–
						>50% increase in *X*_5_	0.0 (0.0–21.5)^¶^	96.0 (88.8–98.9)^¶^	–
						>80% decrease in A*X*	0.0 (0.0–27.8)^¶^	97.2 (90.4–99.5)^¶^	–
						>40% decrease in *R*_5_ or >50% increase in *X*_5_ or >80% decrease in A*X*	0.0 (0.0–22.8)^¶^	95.0 (87.8–98.0)^¶^	–
**Meoli [38] 2024**	72 (63.8)	36 (63.9)	7 (19.4)	5.15*±*0.99^#^	113.14*±*8.9^#^	>25.7% decrease in *R*_5_	71 (36–95)	79 (62–90)	0.77
						>25.7% increase in *X*_5_	86 (49–99)	69 (51–83)	0.75
						>40% decrease in *R*_5_ or >50% increase in *X*_5_ or >80% decrease in A*X*	0.0 (0.0–35.4)^¶^	86.2 (69.4–94.5)^¶^	–

N: total study participants; n: participants completing both spirometric and oscillometric BDR tests. Results reported as mean±sd unless otherwise stated. Values calculated for total study participants unless otherwise stated. Group A: participants with asthma (n=88); Group B: nonasthma participants (n=29); Group a: spirometric BDR positive (n=13); Group b: oscillometric BDR positive (n=4); Group c: negative for both BDRs (n=76). AUROC: area under the receiver operating characteristic curve; A*X*: area of reactance; IQR: interquartile range; NR: not reported; *R*_5_: resistance at 5 Hz; *R*_6_: resistance at 6 Hz; *R*_8_: resistance at 8 Hz; *R*_10_: resistance at 10 Hz; *R*_20_: resistance at 20 Hz; *X*_5_: reactance at 5 Hz. ^#^: Values have been calculated for the participants completing spirometric and oscillometric BDR tests only. ^¶^: Values calculated for this review based on published data or data provided by authors (all other values have been directly extracted from studies).

For BDR testing, Ramirez *et al.* [37] and Meoli *et al.* [38] utilised the diagnostic thresholds of 40% decrease in *R*_5_ or 50% increase in *X*_5_ or 80% decrease in A*X*, recommended in the ERS technical standards [26]. No participants in either study had both a positive oscillometric and spirometric BDR, reporting zero sensitivity and high specificity of these thresholds (table 1). Meoli *et al.* [38] recommended alternative thresholds of a >25.7% decrease in *R*_5_ or a >25.7% increase in *X*_5_, which were shown to have better sensitivity, specificity and AUROC values of ≥0.75.

**FIGURE 3:**
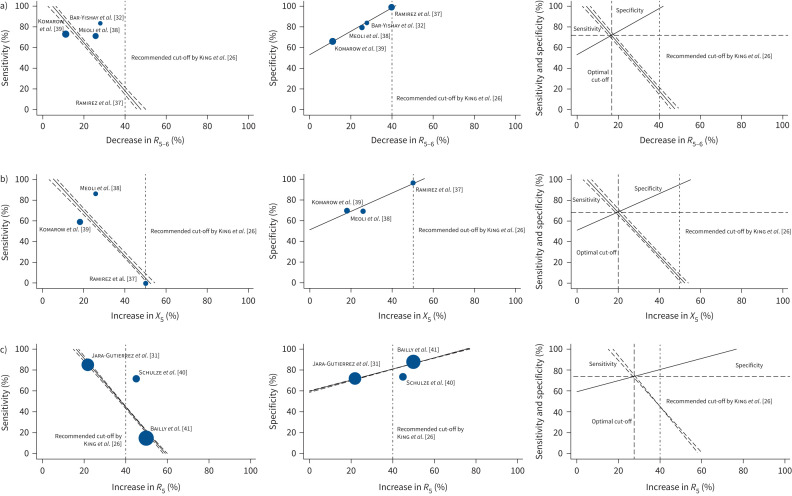
Weighted regression analyses of sensitivity and specificity of studies. a) Studies reporting resistance at 5–6 Hz (*R*_5–6_) for bronchodilator response (BDR). b) Studies reporting reactance at 5 Hz (*X*_5_) for BDR. c) Studies reporting resistance at 5 Hz (*R*_5_) for methacholine challenge test (MCT). The final graphs in each panel show the intersection between sensitivity and specificity, providing the optimal threshold value at equal sensitivity and specificity. The dot-dash line shows the thresholds recommended by King *et al.* [26] in the current European Respiratory Society technical standards.

Due to the significant variation in oscillometry parameters reported by studies, it was impossible to conduct a meta-analysis of the sensitivity and specificity of oscillometry parameters for BDR testing. We instead conducted a *post hoc* weighted regression analysis of the four studies reporting *R*_5_ or *R*_6_ as individual parameters (Bar-Yishay *et al.* [32], Komarow *et al.* [39], Ramirez *et al.* [37] and Meoli *et al.* [38]) to determine a “balanced” BDR threshold that equally optimised both sensitivity and specificity. A 17.0% (95% CI 16.1–17.8%) decrease in *R*_5–6_ was associated with sensitivity and specificity of 71.64% (95% CI 69.7–73.7%) (figure 3). We subsequently conducted a sensitivity analysis, excluding Bar-Yishay *et al.* [32] as this study was at high risk of bias. Results of this analysis identified that a 15.6% (95% CI 14.9–16.2%) decrease in *R*_5_ was associated with optimal sensitivity and specificity of 70.1% (95% CI 68.5–71.8%). Results of additional approaches to determine alternative thresholds including the product, the Youden and 95% specificity approaches [30] are shown in table S4.

Similar weighted regression analysis of the three studies reporting *X*_5_ (Komarow *et al.* [39], Ramirez *et al.* [37] and Meoli *et al.* [38]) as individual parameter showed that a 20.2% (95% CI 19.1–21.1%) increase in *X*_5_ was associated with balanced and optimised sensitivity and specificity of 68.6% (95% CI 66.6–70.8%) (figure 3). Since all studies contributing data to this *X*_5_ analysis were at low risk of bias and of applicability concerns, no sensitivity analysis was conducted. Results of additional approaches to determine alternative thresholds including the product, Youden and 95% specificity approaches [30] are shown in table S4.

*Post hoc* analysis evaluating the dose of salbutamol used for BDR testing was conducted. Weighted regression of the three studies utilising a dose of 200 µg of salbutamol (Bar-Yishay *et al.* [32], Komarow *et al.* [39] and Ramirez *et al.* [37]) showed that a 15.3% (95% CI 14.4–16.2%) decrease in *R*_5–6_ was associated with optimal sensitivity and specificity of 70.4% (95% CI 68.3–72.6%). As Bar-Yishay *et al.* [32] did not report *X*_5_, we were unable to carry out this *post hoc* analysis on salbutamol dose for this parameter.

### Studies evaluating airway hyperresponsiveness

Five studies evaluated the diagnostic accuracy of oscillometry compared to MCT for the diagnosis of asthma.

#### Study characteristics

Studies included only participants under investigation for asthma, except for Jara-Gutierrez *et al.* [31] who included 30 healthy controls in their cohort (of 171 participants). Oscillometry and spirometry devices used, devices used for methacholine administration, and methacholine dosing protocols are summarised in table S3. Jara-Gutierrez *et al.* [31] analysed and reported percentage of predicted normal values for oscillometry and spirometry whilst Schulze *et al.* [40] and Bailly *et al.* [41] reported percentage of predicted normal values for spirometry and raw values for oscillometry. The remaining two studies analysed and reported raw values for oscillometry and spirometry.

Four of five studies measured and reported the provocation dose of methacholine required for a 20% decrease in FEV_1_, the currently recommended standard, whilst one study, Jara-Gutierrez *et al.* [31], measured the provocation concentration.

#### Analyses and results

Demographics of study participants along with reported thresholds for oscillometry parameters and their associated sensitivities and specificities are summarised in table 2. Across all five studies, 503 of 531 participants (94.7%) were able to complete both spirometric and oscillometric MCT. Schulze *et al.* [40] had the youngest participants (mean age 5.3 years) yet 84.2% of them were able to complete both oscillometry and spirometry. Of the 171 participants completing both spirometry and oscillometry in Jara-Gutierrez *et al.* [31], 30 were healthy controls, one of whom had a positive MCT by spirometry.

**TABLE 2:** Results of studies evaluating airway hyperresponsiveness using methacholine challenge test (MCT)

First author [ref.], year	N (% male)	n (% male)	Positive MCT n (%)	Age (years)	Height (cm)	Oscillometry parameter and cut-off value	Sensitivity % (95% CI)	Specificity % (95% CI)	AUROC
**Bouaziz [33] 1996**	38 (44.7)	38 (44.7)	23 (60.5)	9.0*±*0.4	NR	>70% increase in *R*_12_	87	67	–
						>50% increase in *R*_12_	96	40	–
						>1 kPa·L^−1^·s^−1^ decrease in *X*_12_	70	80	–
**Vink [42] 2003**	19 (36.8)	19 (36.8)	14 (73.7)	10.5*±*3.5	146.6*±*19.8	*R*_5_	–	–	0.75
						*R*_10_	–	–	0.68
**Bailly [41] 2011**	227 (55.9)	227 (55.9)	188 (82.8)	9.0*±*3.1	135.7*±*18.3	>50% increase in *R*_5_	14.9 (10.5–20.7)^¶^	87.2 (73.3–94.4)^¶^	0.51
						>50% decrease in *X*_5_	36.2 (29.6–43.3)^¶^	84.6 (70.3–92.8)^¶^	0.61
**Schulze [40] 2012**	57 (NR)	48 (60.4)	40 (83.3)	5.3*±*0.9^#^	114.9*±*7.9^#^	>40% increase in *R*_5_	70.0 (54.6–81.9)^¶^	37.5 (13.7–69.4)^¶^	–
						>45.2% increase in *R*_5_	72	73	0.76
						>0.69 kPa·L^−1^·s^−1^ decrease in *X*_5_	80	76	0.81
						>45% increase in *R*_5_ OR >0.69 kPa·L^−1^·s^−1^ decrease in *X*_5_	74	76	–
**Jara-Gutierrez [31] 2019**	190 (55.2)	171 (NR)	132 (77.2)	Median*±*sd 10.0*±*3.1	Median*±*sd 140.8*±*17.5	>22% increase in *R*_5_	85	71	0.89
						>41% decrease in *X*_5_	87	69	0.88
						>82% increase in A*X*	83	78	0.86

N: total study participants; n: participants completing both spirometric and oscillometric MCT. Results reported as mean±sd unless otherwise stated. All values calculated for total study participants unless otherwise stated. AUROC: area under the receiver operating characteristic curve; A*X*: area of reactance; NR: not reported; *R*_5_: resistance at 5 Hz; *R*_10_: resistance at 10 Hz; *R*_12_: resistance at 12 Hz; *R*_20_: resistance at 20 Hz; *X*_5_: reactance at 5 Hz; *X*_12_: reactance at 12 Hz. ^#^: Values have been calculated for the participants completing spirometric and oscillometric MCT only. ^¶^: Values calculated for this review based on published data or data provided by authors (all other values have been directly extracted from studies).

Bailly *et al.* [41] recommended a threshold of >50% increase in *R*_5_, but this had low sensitivity, high specificity and low AUROC values. Schulze *et al.* [40] recommended a slightly lower threshold of a >45.2% increase in *R*_5_, which demonstrated better sensitivity, specificity and AUROC. Jara-Gutierrez *et al.* [31] recommended a >22% increase in *R*_5_ and this showed a higher sensitivity with slightly lower specificity and AUROC values of >0.85.

Due to the significant variation in oscillometry parameters reported, a meta-analysis of oscillometry was not possible for MCT. As *post hoc* analysis, we conducted a weighted regression analysis of the only three studies reporting *R*_5_ (Bailly *et al.* [41], Schulze *et al.* [40] and Jara-Gutierrez *et al.* [31]) in their study population. The results of this analysis showed that a 27.7% (95% CI 27.0–28.4%) increase in *R*_5_ would be an optimal threshold for determining airway hyperresponsiveness. This has a balanced sensitivity and specificity of 73.6% (95% CI 71.9–75.3%) (figure 3). Results of additional approaches to determine ideal thresholds including the product, Youden and 95% specificity approaches [30] are shown in table S4. As only three studies were included in this analysis, no sensitivity analyses were possible.

## Discussion

Respiratory oscillometry measured during normal tidal breathing is attracting increasing interest as a noninvasive clinical tool in the asthma diagnostic pathway in children and technical standards and thresholds have been published recently [26]. Oscillometry is feasible in young children, with more than 85% of participants across all studies being able to complete the test. Our review comprehensively summarises the current literature on oscillometry *versus* spirometry using BDR or MCT for diagnosing asthma in children.

Of the six included studies evaluating BDR, Ramirez *et al.* [37] and Meoli *et al.* [38] utilised the diagnostic thresholds recommended in the 2020 ERS technical guidelines [26]. Using this approach was associated with a high specificity ranging between 86 and 95% but zero sensitivity (confidence interval ranging from 0–35%). These findings are derived from relatively small studies (<100 participants with <20% of them displaying positive BDR by spirometry), which may explain the wide confidence interval around these estimates. Yet the findings indicate that recommended oscillometry thresholds are insensitive and result in the nonidentification of many more children with asthma than use of the single spirometry FEV_1_ threshold of ≥12%. These recommended threshold values were based on the upper limit of BDR in healthy children [26], which while specific, may not be the best population from which to derive thresholds to apply to children under investigation for suspected asthma. Interestingly, in a recent Delphi study, there was no consensus between expert clinicians on oscillometry BDR thresholds for diagnosing asthma in adults [43].

Our data suggests that alternative oscillometry BDR threshold values are needed for the diagnosis of asthma in children. Our suggested data-derived thresholds for *R*_5_ and *X*_5_ are significantly lower than those recommended by the ERS technical guidelines [26]. It is important to note that the oscillometry BDR thresholds we calculated based on existing data, optimised for sensitivity and specificity in this systematic review, could still result in approximately one-third of children being under- (false negative) and overdiagnosed (false positive) using these cut-offs. This rate of misdiagnosis is problematic and children would almost certainly require additional tests. Using oscillometry as part of a composite diagnostic approach merits further evaluation.

In adults, many studies have investigated the usefulness of impulse oscillometry (pre- and post-bronchodilator) in supporting spirometry for the diagnosis of asthma [44–48]. Comparisons between spirometry BDR and oscillometry BDR have also been published for adult patients with moderate to severe asthma; however, the oscillometry cut-offs used were different [49, 50] to those proposed in the current ERS technical standards [26].

A positive spirometric BDR is widely considered the gold standard diagnostic test for asthma, yet the literature consistently reports a relatively low sensitivity (35–36%) and high specificity (90–98%) for a 12% or greater change in FEV_1_ [51, 52]. For our review, we aimed to maximise both sensitivity and specificity, allowing the oscillometry threshold to be as accurate as possible. Given that a higher threshold will result in lower sensitivity and higher specificity, and *vice versa*, a balanced threshold equally optimising sensitivity and specificity was proposed. Whilst the weighted regression analysis allowed the study sample sizes to be included and thus reduced uncertainty in model predictions, more studies focusing on *R*_5_ would be desirable for inclusion in future updates. This is especially pertinent given the heterogeneity between studies.

Of five studies that assessed airway hyperresponsiveness using the MCT, three reported *R*_5_ and our weighted regression analysis allowed the identification of an optimal threshold, equally optimising sensitivity and specificity. Further work is required for prospective validation of this threshold. Of note, the ERS technical standards for oscillometry [26] did not include a recommendation for MCT thresholds.

The focus of our review has been on BDR and MCT using oscillometry; however, baseline oscillometry (without additional testing) could also be useful to identify children with asthma. Ramirez *et al.* [37] reported that participants who had a positive spirometric BDR had significantly higher *X*_5_ at baseline compared to those who were negative for both BDRs. Meoli *et al.* [38] reported that participants who had a positive spirometric BDR had a higher *R*_5_ and more negative *X*_5_ at baseline than their counterparts but, the differences were not significant. Similarly, Komarow *et al.* [39] reported no significant difference in baseline oscillometry and spirometry values between asthma and nonasthma groups. Of the five studies evaluating MCT in our review, only Schulze *et al.* [40] reported baseline values and reported no significant differences in oscillometry and spirometry parameters at baseline between participants with a positive MCT compared to the total group. Further work is thus required to determine if baseline oscillometry values could be useful for identifying children with asthma alone or in combination with BDR. Population z-scores for oscillometry parameters in healthy children have been published and are valuable in this context [53].

### Strengths and limitations

Strengths of our review include that we carried out a comprehensive literature search up to March 2025 and rigorously evaluated the quality of included studies using the QUADAS-2 tool [29]. We only included studies that used well-established, objective tests as the reference standard (spirometry with BDR or airway hyperresponsiveness assessed by MCT). We utilised weighted regression analyses to suggest optimal thresholds (giving equal sensitivity and specificity) for oscillometry parameters for BDR and MCT. Our graphs in figure 3 and additional analyses shown in table S4 allow readers to consider alternative thresholds, depending on the clinical context; for example, maximising sensitivity to reduce the risk of false negatives or maximising specificity to reduce the risk of a false-positive diagnosis.

The data were limited by the variation in oscillometry parameters reported by studies, which meant meta-analyses were not possible. We contacted authors of all included studies for raw data but only received two positive responses out of 11; thus, individual patient data meta-analyses and primary ROC curve analyses were not possible.

Oscillometry measures tidal breathing whilst spirometry utilises forced expiratory manoeuvres; however, a recent study in adults showed that the oscillometric response to methacholine is significantly attenuated by concomitant spirometry [54]. Using oscillometry alone also reduces the number of doubling concentrations needed to reach an equivalent response [54]. This is a limitation of the MCT studies in our review. Although all studies (except Bouaziz *et al.* [33]) report systematically measuring oscillometry prior to spirometry, the extent by which subsequent oscillometric readouts are influenced by prior spirometric manoeuvres is unclear. Future studies evaluating oscillometry thresholds for MCT should consider whether testing on separate days is needed to remove the effect of this attenuation.

We compared the sensitivity and specificity of oscillometry to either a positive BDR or positive MCT; however, the diagnosis of asthma is rarely based on a single test. Future studies should evaluate how oscillometry would compare to, or perform in combination with, alternative tests such as *F*_ENO_ or within a robust diagnostic algorithm for asthma, as recommended by GINA [8].

Considering the generalisability of our findings, most studies in our review included school-age children (>6 years) and it remains unclear whether findings could be extrapolated to the preschool (2–5 years) age group. Prospective validation is required to determine whether the same diagnostic thresholds can be applied to preschoolers or whether separate oscillometry thresholds are required.

### Questions for future research

Further research is required to prospectively validate our proposed thresholds for BDR and MCT in children under investigation for asthma. To ensure that prospective data is reproducible and meta-analysable, a consistent framework for measuring and reporting oscillometry parameters is required. Across studies included in our review, *R*_5_ and *X*_5_ were the most commonly reported oscillometry parameters, followed by *R*_10_ and A*X*. We recommend that future studies evaluating oscillometry should measure and report these four parameters, as well as *R*_5–19_ or *R*_7–19_, ideally. Moreover, consistency of measurement accuracy between devices is highly desired [55, 56]. Technical standards for data acquisition of oscillometry have been recently published by the ERS [26] and recommend the reporting of z-scores in addition to raw values. Validated device-specific reference equations for calculating oscillometry z-scores in children have also been recently published [53] and should be applied to report z-scores. Open research principles should be promoted, allowing for individual patient meta-analyses in future.

Oscillometry is feasible in young children and has the potential to be a valuable diagnostic tool. Our systematic evaluation of existing literature has shown variation across studies in reported oscillometry parameters, methodological quality of respiratory oscillometry measurements and the technical acceptability of results. Further work needs to be done to establish the diagnostic accuracy of oscillometry and to validate diagnostic thresholds for adoption in clinical practice.

## Conclusion

This review comprehensively summarises the current literature on oscillometry. Our findings suggest that currently recommended ERS thresholds for BDR (>40% decrease in *R*_5_, >50% increase in *X*_5_ or >80% decrease in A*X*) have very low sensitivity. Alternative oscillometry BDR thresholds are required for the diagnosis of asthma in children and young people. Based on our weighted regression analyses, a 17.0% decrease in *R*_5–6_ (with equal sensitivity and specificity of 71.64%, 95% CI 69.7–73.7%) or a 20.2% increase in *X*_5_ (with equal sensitivity and specificity of 68.6%, 95% CI 66.6–70.8%) following bronchodilator were optimal thresholds to define positive BDR. For positive MCT, the optimal threshold was a 27.7% increase in *R*_5_ (with equal sensitivity and specificity of 73.6%, 95% CI 71.9–75.3%). However, despite all the available data, these thresholds could still result in a significant rate of misdiagnosis. Further well-designed prospective studies are needed to establish the asthma diagnostic accuracy of oscillometry and define BDR and MCT diagnostic thresholds in preschoolers and school-age children for use as a standalone test or alongside other tests within a diagnostic algorithm. We highlight the need for consistency in measuring and reporting oscillometry parameters in future studies.

## Supplementary material

**Please note:** supplementary material is not edited by the Editorial Office, and is uploaded as it has been supplied by the author. Supplementary data tables ERR-0293-2025.SUPPLEMENT Supplementary appendix 1: search strategy ERR-0293-2025.SUPPLEMENT2
