# Supplementary material for: Oscillometry for the diagnosis of asthma in children: a systematic review
Source: Eur Respir Rev. 2026 Apr 29;35(180):250293. doi: 10.1183/16000617.0293-2025 (PMC13126124; doi:10.1183/16000617.0293-2025)
Supplement: Supplementary file 1 [file ERR-0293-2025.SUPPLEMENT.pdf]

SUPPLEMENTARY DATA

Supplementary Table 1. Summary of risk of bias assessment of all included studies utilising the QUADAS-2 tool. [29]

	Study	Risk of Bias				Concerns regarding Applicability		
		Patient selection	Index test	Reference standard	Flow and timing	Patient selection	Index test	Reference standard
Studies evaluating BDR	Bar Yishay 2009	Unclear risk	High risk	High risk	Unclear risk	Low risk	Low risk	Low risk
	Komarow 2012	Low risk	Low risk	Low risk	Low risk	Low risk	Low risk	Low risk
	Sheen 2018	Unclear risk	Low risk	Low risk	Low risk	Low risk	Low risk	Low risk
	Lauhkonen 2021	Low risk	Unclear risk	Low risk	Unclear risk	Low risk	Low risk	Low risk
	Ramirez 2021	Low risk	Low risk	Low risk	Low risk	Low risk	Low risk	Low risk
	Meoli 2024	Low risk	Low risk	Low risk	Low risk	Low risk	Low risk	Low risk
Studies evaluating MCT	Bouaziz 1996	Low risk	Low risk	Low risk	High risk	High risk	High risk	Low risk
	Vink 2003	Low risk	Unclear risk	Low risk	Low risk	Low risk	Low risk	Low risk
	Bailly 2011	Unclear risk	Unclear risk	Low risk	Low risk	Low risk	Low risk	Low risk
	Schulze 2012	Unclear risk	Low risk	Low risk	Low risk	Low risk	Low risk	Low risk
	Jara Gutierrez 2019	High risk	Low risk	Low risk	Low risk	Low risk	Low risk	Low risk

■ High risk

□ Low risk

▨ Unclear risk

Supplementary Table 2. Summary of oscillometry and spirometry devices used and bronchodilator doses administered across studies. Abbreviations: coherence at 5Hz (COH(5)); coherence at 10Hz (COH(10)); coherence at 20Hz (COH(20)); coefficient of variation (CV); resistance at 5Hz (R5); resistance at 20Hz (R20).

STUDY	DEVICE USED		BRONCHODILATOR ADMNISTERED	QUALITY CONTROL FOR OSCILLOMETRY
	Oscillometry	Spirometry		
**Bar Yishay 2009**	Chess i2m system<br>(Chess mt NV, Oostakker, Belgium)	Zan system<br>(Zan 100, nSpire Health Inc., Germany)	Salbutamol<br>200 micrograms	- 8s acquisition time
**Komarow 2012**	MasterScreen device<br>(CareFusion, Yorba Linda, CA, USA)	Vmax Encore Model 20C<br>(Care Fusion, Yorba Linda, CA, USA)	Salbutamol<br>180 micrograms	- 30s acquisition time<br>- 3 measurements with COH(10) >0.8
**Sheen 2018**	MasterScreen device<br>(Jaeger Co, Wurzburg, Germany)	MasterScreen device<br>(Jaeger Co, Wurzburg, Germany)	Salbutamol<br>400 micrograms	- 30s acquisition time<br>- 3 acceptable measurements
**Lauhkonen 2021**	Resmon Pro device<br>(MGC Diagnostics, USA)	EasyOne Pro Lab device<br>(NDD, Switzerland)	Salbutamol<br>400 micrograms	Not reported
**Ramirez 2021**	MasterScreen device<br>(Jaeger Co, Wurzburg, Germany)	Vyntus SPIRO device<br>(Jaeger Co, Wurzburg, Germany)	Salbutamol<br>200 micrograms	- 8-16s acquisition time<br>- 3-5 measurements with CV <10% for R5 and R20 and COH(5) >0.6
**Meoli 2024**	Vyntus APS/IOS device (Vyaire GmbH, Germany)	Vyntus APS/IOS device (Vyaire GmbH, Germany)	Salbutamol<br>400 micrograms	- 20s acquisition time<br>- 3 artefact-free measurements with COH(5)≥0.8, COH(20) ≥0.9 and CV≤15%

Supplementary Table 3. Summary of oscillometry and spirometry devices used and methacholine dosing protocols across studies. Abbreviations: coefficient of variation (CV); resistance at 5Hz (R5).

STUDY	DEVICE USED		MAX CUMULATIVE DOSE OF METHACHOLINE	QUALITY CONTROL FOR OSCILLOMETRY
	Oscillometry	Spirometry		
**Bouaziz 1996**	Pulmosfor<br>(SEFAM, France)	Flow meter<br>(Spiromatic, Gauthier, France)	1200 micrograms	- 10s acquisition time<br>- 2 measurements with coherence >0.95
**Vink 2003**	Masterlab-IOS device<br>(Jaeger Co, Wurzburg, Germany)	MasterScreen device<br>(Jaeger Co, Wurzburg, Germany)	1565 micrograms	- 60s acquisition time
**Bailly 2011**	MasterScreen device<br>(Jaeger Co, Wurzburg, Germany)	Autospiro PAL device<br>(Minato Medical, Japan)	1500 micrograms	- 30s acquisition time
**Schulze 2012**	Impulse Oscillometry System<br>(CareFusion, Hoechberg, Germany)	MasterScreen device<br>(CareFusion, Hoechberg, Germany)	2900 micrograms	- 20s acquisition time<br>- Minimum 3 acceptable measurements
**Jara Gutierrez 2019**	MasterScreen device<br>(Jaeger Co, Wurzburg, Germany)	MasterScreen device<br>(Jaeger Co, Wurzburg, Germany)	Concentration range used<br>0.125mg/ml - 16mg/ml	- 30s acquisition time<br>- 3 measurements with CV <6% for R5

Supplementary Table 4. Results of alternative approaches to determine an ideal diagnostic threshold. Abbreviations: bronchodilator response (BDR); methacholine challenge test (MCT); resistance at 5Hz or 6Hz (R5-6); reactance at 5Hz (X5); resistance at 5Hz (R5); 95% confidence interval (95% CI); area under receiver operating characteristic curve (AUROC) .

A) R5-6 BDR threshold

Methods	Cut-off (95% CI)	Sensitivity (95% CI)	Specificity (95% CI)	AUROC
Proposed	17.0 (16.1-17.8)	71.6 (69.7-73.7)	71.6 (69.7-73.7)	71.6
Product	4.8 (.-18.7)	100 (.)	57.9 (53.9-61.9)	79.0
Youden	4.8 (.-18.7)	100 (.)	57.9 (53.9-61.9)	79.0
95% Specificity	37.8 (35.4-40.7)	23.6 (0.0-66.3)	95 (.)	59.3

B) X5 BDR threshold

Methods	Cut-off (95% CI)	Sensitivity (95% CI)	Specificity (95% CI)	AUROC
Proposed	20.2 (19.1-21.1)	68.6 (66.6-70.8)	68.6 (66.6-70.8)	68.6
Product	5.3 (.-23.5)	100 (.)	55.4 (44.4-66.4)	77.7
Youden	5.3 (.-23.5)	100 (.)	55.4 (44.4-66.4)	77.7
95% Specificity	49.8 (42.1-66.2)	5.7 (0.0-64.0)	95 (.)	50.4

C) R5 MCT threshold

Methods	Cut-off (95% CI)	Sensitivity (95% CI)	Specificity (95% CI)	AUROC
Proposed	27.7 (27.0-28.4)	73.6 (71.9-75.3)	73.6 (71.9-75.3)	73.6
Product	16.4 (.-30.2)	100 (.)	67.5 (54.1-80.9)	83.8
Youden	16.4 (.-30.2)	100 (.)	67.5 (54.1-80.9)	83.8
95% Specificity	67.8 (50.7-.)	0.0 (0.0-45.2)	95 (.)	47.5

Methods

Proposed method = jointly maximising sensitivity and specificity

*Product = maximising (sensitivity*specificity)*

Youden = maximising (sensitivity+specificity-1)

AUROC = area under ROC curve, calculated for each method based on cut-point. Please note AUROC values have been approximated as no individual patient data was available for analysis.

Interpretation

Our proposed method gives reasonable balance between sensitivity and specificity. Product and Youden prefer high sensitivity as this decreases more quickly than specificity increases; thus optimum cut-point is found at sensitivity = 100%. No LCI for cut-offs under product/Youden methods as LCI for sensitivity does not reach 100%. No LCI or UCI for sensitivity under product/Youden methods as sensitivity/specificity is maximised at sensitivity = 100% under these methods. AUC is slightly better for product and Youden methods than our proposed method due to high sensitivity.

Cut-offs for 95% specificity are also reported, indicate poor sensitivity (but unsurprising as we are prioritising specificity). No UCI for cut-off under MCT R5 as LCI for specificity does not reach 95%. Area under ROC curve is poor under this method due to poor sensitivity.

Supplementary Table 5. Results of alternative approaches to determine an ideal diagnostic threshold for BDR sensitivity analyses. Abbreviations: bronchodilator response (BDR); methacholine challenge test (MCT); resistance at 5Hz or 6Hz (R5-6); reactance at 5Hz (X5); resistance at 5Hz (R5); 95% confidence interval (95% CI); area under receiver operating characteristic curve (AUROC) .

a) BDR sensitivity analysis for R5-6, excluding Bar-Yishay 2009[32] (as high risk of bias)

Methods	Cut-off (95% CI)	Sensitivity (95% CI)	Specificity (95% CI)	AUROC
Proposed	15.6 (14.9-16.2)	70.1 (68.5-71.8)	70.1 (68.5-71.8)	70.1
Product	3.4 (.-17.3)	100 (.)	56.5 (50.6-62.3)	78.3
Youden	3.4 (.-17.3)	100 (.)	56.5 (50.6-62.3)	78.3
95% Specificity	37.6 (34.3-42.2)	16.0 (0.0-59.3)	95 (.)	55.5

b) BDR sensitivity analysis for R5-6, including only studies using 200 micrograms salbutamol (excluding Meoli 2024[38])

Methods	Cut-off (95% CI)	Sensitivity (95% CI)	Specificity (95% CI)	AUROC
Proposed	15.3 (14.4-16.2)	70.4 (68.3-72.6)	70.4 (68.3-72.6)	70.4
Product	2.7 (.-20.4)	100 (.)	56.2 (53.7-58.8)	78.1
Youden	2.7 (.-20.4)	100 (.)	56.2 (53.7-58.8)	78.1
95% Specificity	37.1 (35.6-38.8)	19.2 (0.0-73.7)	95 (.)	57.1
