# Supplementary material for: Oscillometry for the diagnosis of asthma in children: a systematic review
Source: Eur Respir Rev. 2026 Apr 29;35(180):250293. doi: 10.1183/16000617.0293-2025 (PMC13126124; doi:10.1183/16000617.0293-2025)
Supplement: Supplementary file 2 [file ERR-0293-2025.SUPPLEMENT2.pdf]

APPENDIX 1 – SEARCH STRATEGY

Ovid MEDLINE(R)

1 exp Asthma/
2 asthma*.ti,ab,kw,kf.
3 Respiratory Sounds/
4 Dyspnea/
5 (wheez* or rhonchi or cough* or breathless* or dyspn?e*).ti,ab,kw,kf.
6 ((difficult* or labo?r* or short*) adj2 breath*).ti,ab,kw,kf.
7 Bronchial Hyperreactivity/
8 airway hyperresponsiveness.ti,ab,kw,kf.
9 or/1-8
10 Oscillometry/
11 Airway Resistance/
12 ((airway or respirat*) adj2 (impedance or reactance or resistance)).ti,ab,kw,kf.
13 ((forced or impulse or sinusoidal) adj2 oscill*).ti,ab,kw,kf.
14 (TremoFlo or Resmon or Jaeger).mp.
15 or/10-14
16 vital capacity/
17 forced expiratory volume/
18 (FEV1 or FEV 1 or FVC).ti,ab,kw,kf.
19 (flow volume adj (loop* or curve* or graph*)).ti,ab,kw,kf.
20 (forced expiratory volume* adj6 ("1" or one)).ti,ab,kw,kf.
21 ((force* or time*) adj vital capacit*).ti,ab,kw,kf.
22 spirometr*.ti,ab,kw,kf.
23 PEFV.ti,ab,kw,kf.
24 (PEF or PEFR or PFR or peak expiratory flow* or peak flow*).ti,ab,kw,kf.
25 Peak Expiratory Flow Rate/
26 Bronchial Provocation Tests/
27 (Methacholine adj2 challenge*).mp. [mp=title, book title, abstract, original title, name of substance word, subject heading word, floating sub-heading word, keyword heading word, organism supplementary concept word, protocol supplementary concept word, rare disease supplementary concept word, unique identifier, synonyms, population supplementary concept word, anatomy supplementary concept word]
28 bronchoprovocation.mp.
29 or/16-28
30 (babies or baby or infan* or neonat* or neo-nat* or newborn* or new-born* or perinat*).ti,ab,kf. or Infant, Extremely Low Birth Weight/ or Infant, Extremely Premature/ or Infant, Large for Gestational Age/ or Infant, Low Birth Weight/ or Infant, Newborn/ or Infant, Postmature/ or Infant, Premature/ or Infant, Small for Gestational Age/ or Infant, Very Low Birth Weight/ or Infant/ or (boy? or boyfrien* or boyhood* or child* or fifth-grader* or first-grader* or fourth-grader* or girl? or girlfriend* or girlhood* or juvenil* or kid? or kindergarten* or minor? or minority or paediatric* or peadiatric* or pediatric* or PICU or preschool* or pre-school* or second-grader* or seventh-grader* or sixth-grader* or stepchild* or step-child* or third-grader* or toddler? or young or youngster* or youth*).ti,ab,kf. or Child/ or Child, Preschool/ or (adolescen* or college* or highschool* or high-school* or ((high* or

secondary) adj2 (education or school*)) or preadolescen* or pre-adolescen* or preteen* or pre-teen* or puber* or pubescen* or teen* or underage? or under-age?).ti,ab,kf. or Adolescent/ or Minors/ or (young adj2 (adult* or individual* or inpatient* or in-patient* or man or men or outpatient* or out-patient* or patient* or people* or person* or population* or wom#n)).ti,ab,kf. or Young Adult/
31 9 and 15 and 29 and 30

Embase

1 exp asthma/
2 asthma*.ti,ab,kw,kf.
3 abnormal respiratory sound/
4 dyspnea/
5 (wheez* or rhonchi or cough* or breathless* or dyspn?e*).ti,ab,kw,kf.
6 ((difficult* or labo?r* or short*) adj2 breath*).ti,ab,kw,kf.
7 bronchus hyperreactivity/
8 airway hyperresponsiveness.mp. or bronch* hyperreactivity.ti,ab,kw,kf. [mp=title, abstract, heading word, drug trade name, original title, device manufacturer, drug manufacturer, device trade name, keyword heading word, floating subheading word, candidate term word]
9 respiratory tract allergy/
10 or/1-9
11 impulse oscillometry/ or oscillometry/
12 airway resistance/
13 ((airway or respirat*) adj2 (impedance or reactance or resistance)).ti,ab,kw,kf.
14 ((forced or impulse or sinusoidal) adj2 oscill*).ti,ab,kw,kf.
15 (jaeger or resmon or TremoFlo).mp. [mp=title, abstract, heading word, drug trade name, original title, device manufacturer, drug manufacturer, device trade name, keyword heading word, floating subheading word, candidate term word]
16 forced oscillation technique/
17 or/11-16
18 vital capacity/
19 forced expiratory volume/
20 (FEV1 or FEV 1 or FVC).ti,ab,kw,kf.
21 (flow volume adj (loop* or curve* or graph*)).ti,ab,kw,kf.
22 (forced expiratory volume* adj6 ("1" or one)).ti,ab,kw,kf.
23 ((force* or time*) adj vital capacit*).ti,ab,kw,kf.
24 spirometr*.ti,ab,kw,kf.
25 spirometry/
26 PEFV.ti,ab,kw,kf.
27 (PEF or PEFR or PFR or peak expiratory flow* or peak flow*).ti,ab,kw,kf.
28 peak expiratory flow/
29 inhalation test/
30 Bronchial Provocation Test*.mp.
31 (Methacholine adj2 challenge*).mp. [mp=title, abstract, heading word, drug trade name, original title, device manufacturer, drug manufacturer, device trade name, keyword heading word, floating subheading word, candidate term word]
32 methacholine/ and provocation test/
33 bronchoprovocation.mp.
34 or/18-33

35 exp adolescence/ or exp adolescent/ or exp child/ or exp childhood disease/ or exp infant disease/ or (adolescen* or babies or baby or boy? or boyfriend or boyhood or girlfriend or girlhood or child* or girl? or infan* or juvenil* or juvenile* or kid? or minors or minors* or neonat* or neonat* or neo-nata* or newborn* or new-born* or paediatric* or peadiatric* or pediatric* or perinat* or preschool* or puber* or pubescen* or school or school child* or school* or schoolchild* or schoolchild* or teen* or toddler? or underage? or under-age? or youth*).ti,ab,kw.
36 10 and 17 and 34 and 35

Ovid Emcare

1 exp asthma/
2 asthma*.ti,ab,kw,kf.
3 abnormal respiratory sound/
4 dyspnea/
5 (wheez* or rhonchi or cough* or breathless* or dyspn?e*).ti,ab,kw,kf.
6 ((difficult* or labo?r* or short*) adj2 breath*).ti,ab,kw,kf.
7 bronchus hyperreactivity/
8 airway hyperresponsiveness.mp. or bronch* hyperreactivity.ti,ab,kw,kf. [mp=title, abstract, heading word, drug trade name, original title, device manufacturer, drug manufacturer, device trade name, keyword heading word]
9 respiratory tract allergy/
10 or/1-9
11 impulse oscillometry/ or oscillometry/
12 airway resistance/
13 ((airway or respirat*) adj2 (impedance or reactance or resistance)).ti,ab,kw,kf.
14 ((forced or impulse or sinusoidal) adj2 oscill*).ti,ab,kw,kf.
15 (jaeger or resmon or TremoFlo).mp. [mp=title, abstract, heading word, drug trade name, original title, device manufacturer, drug manufacturer, device trade name, keyword heading word]
16 forced oscillation technique/
17 or/11-16
18 vital capacity/
19 forced expiratory volume/
20 (FEV1 or FEV 1 or FVC).ti,ab,kw,kf.
21 (flow volume adj (loop* or curve* or graph*)).ti,ab,kw,kf.
22 (forced expiratory volume* adj6 ("1" or one)).ti,ab,kw,kf.
23 ((force* or time*) adj vital capacit*).ti,ab,kw,kf.
24 spirometr*.ti,ab,kw,kf.
25 spirometry/
26 PEFV.ti,ab,kw,kf.
27 (PEF or PEFR or PFR or peak expiratory flow* or peak flow*).ti,ab,kw,kf.
28 peak expiratory flow/
29 inhalation test/
30 Bronchial Provocation Test*.mp.
31 (Methacholine adj2 challenge*).mp. [mp=title, abstract, heading word, drug trade name, original title, device manufacturer, drug manufacturer, device trade name, keyword heading word]
32 methacholine/ and provocation test/

33 bronchoprovocation.mp.
34 or/18-33
35 exp adolescence/ or exp adolescent/ or exp child/ or exp childhood disease/ or exp infant disease/ or (adolescen* or babies or baby or boy? or boyfriend or boyhood or girlfriend or girlhood or child* or girl? or infan* or juvenil* or juvenile* or kid? or minors or minors* or neonat* or neonat* or neo-nata* or newborn* or new-born* or paediatric* or peadiatric* or pediatric* or perinat* or preschool* or puber* or pubescen* or school or school child* or school* or schoolchild* or schoolchild* or teen* or toddler? or underage? or under-age? or youth*).ti,ab,kw.
36 10 and 17 and 34 and 35

CINAHL

#	Query
S1	(MH "Asthma+")
S2	TI asthma* OR AB asthma*
S3	(MH "Respiratory Sounds")
S4	(MH "Dyspnea")
S5	TI (wheez* or rhonchi or cough* or breathless* or dyspn?e*) OR AB (wheez* or rhonchi or cough* or breathless* or dyspn?e*)
S6	TI (((difficult* or labo?r* or short*) n2 breath*)) OR AB (((difficult* or labo?r* or short*) n2 breath*))
S7	TI Bronchial Hyperreactivity OR AB Bronchial Hyperreactivity
S8	TI airway hyperresponsiveness OR AB airway hyperresponsiveness
S9	S1 OR S2 OR S3 OR S4 OR S5 OR S6 OR S7 OR S8
S10	(MH "Airway Resistance")
S11	TI (((airway or respirat*) n2 (impedance or reactance or resistance))) OR AB (((airway or respirat*) n2 (impedance or reactance or resistance)))
S12	TI (((forced or impulse or sinusoidal) n2 oscill*)) OR AB (((forced or impulse or sinusoidal)

	n2 oscill*))
S13	TI (TremoFlo or Resmon or Jaeger) OR AB (TremoFlo or Resmon or Jaeger)
S14	TI oscillomet* OR AB oscillomet*
S15	S10 OR S11 OR S12 OR S13 OR S14
S16	(MH "Vital Capacity")
S17	(MH "Forced Expiratory Volume")
S18	TI (FEV1 or FEV 1 or FVC) OR AB (FEV1 or FEV 1 or FVC)
S19	TI (flow volume n1 (loop* or curve* or graph*)) OR AB (flow volume n1 (loop* or curve* or graph*))
S20	TI ((forced expiratory volume* n6 ("1" or one))) OR AB ((forced expiratory volume* n6 ("1" or one)))
S21	TI (((force* or time*) n1 vital capacit*)) OR AB (((force* or time*) n1 vital capacit*))
S22	TI spirometr* OR AB spirometr*
S23	(MH "Spirometry")
S24	TI (PEF or PEFR or PFR or peak expiratory flow* or peak flow* or PEFV) OR AB (PEF or PEFR or PFR or peak expiratory flow* or peak flow* or PEFV)
S25	(MH "Peak Expiratory Flow Rate")
S26	(MH "Bronchial Provocation Tests")
S27	TI Methacholine n2 challenge* OR AB Methacholine n2 challenge*
S28	TI bronchoprovocation OR AB bronchoprovocation
S29	S16 OR S17 OR S18 OR S19 OR S20 OR S21 OR S22 OR S23 OR

	S24 OR S25 OR S26 OR S27 OR S28
S30	S9 AND S15 AND S29
S31	(mh "child+") or (mh "adolescence+") or (mh "pediatrics+")
S32	TI child* OR AB child*
S33	TI (infant or baby or babies) OR AB (infant or baby or babies)
S34	TI adolescen* OR AB adolescen*
S35	TI (pediatric* or paediatric*) OR AB (pediatric* or paediatric*)
S36	TI (neonat* or newborn*) OR AB (neonat* or newborn*)
S37	S31 OR S32 OR S33 OR S34 OR S35 OR S36
S38	S30 AND S37

Search Name: Impulse oscillometry
Comment: Cochrane CENTRAL

ID	Search	Hits
#1	MeSH descriptor: [Asthma] explode all trees	
#2	(asthma*):ti,ab,kw	
#3	MeSH descriptor: [Respiratory Sounds] explode all trees	
#4	MeSH descriptor: [Dyspnea] explode all trees	
#5	(wheez* or rhonchi or cough* or breathless* or dyspn?e*):ti,ab,kw	
#6	(((difficult* or labo?r* or short*) near/2 breath*)):ti,ab,kw	
#7	MeSH descriptor: [Bronchial Hyperreactivity] explode all trees	
#8	(airway hyperresponsiveness):ti,ab,kw	
#9	{or #1-#8}	
#10	MeSH descriptor: [Oscillometry] explode all trees	
#11	MeSH descriptor: [Airway Resistance] explode all trees	
#12	(((airway or respirat*) near/2 (impedance or reactance or resistance))):ti,ab,kw	
#13	(((forced or impulse or sinusoidal) near/2 oscill*)):ti,ab,kw	
#14	(TremoFlo or Resmon or Jaeger):ti,ab,kw	
#15	{or #10-#14}	
#16	MeSH descriptor: [Vital Capacity] explode all trees	
#17	MeSH descriptor: [Forced Expiratory Volume] explode all trees	
#18	(FEV1 or FEV 1 or FVC):ti,ab,kw	
#19	(flow volume near/1 (loop* or curve* or graph*)):ti,ab,kw	
#20	(forced expiratory volume* near/6 ("1" or one)):ti,ab,kw	

#21 (((force* or time*) next vital capacit*)):ti,ab,kw
#22 (spirometr*):ti,ab,kw
#23 MeSH descriptor: [Spirometry] explode all trees
#24 (PEF or PEFR or PFR or peak expiratory flow* or peak flow* or PEFV):ti,ab,kw
#25 MeSH descriptor: [Peak Expiratory Flow Rate] explode all trees
#26 MeSH descriptor: [Bronchial Provocation Tests] explode all trees
#27 (Methacholine near/2 challenge*):ti,ab,kw
#28 (bronchoprovocation):ti,ab,kw
#29 {or #16-#28}
#30 MeSH descriptor: [Child] explode all trees
#31 MeSH descriptor: [Adolescent] explode all trees
#32 MeSH descriptor: [Pediatrics] explode all trees
#33 (child*):ti,ab,kw
#34 (infant or baby or babies):ti,ab,kw
#35 (adolescen* or teen*):ti,ab,kw
#36 (neonat* or newborn*):ti,ab,kw
#37 {or #30-#36}
#38 #9 and #15 and #29 and #37
